# Mortalidade por Infarto Agudo do Miocárdio no Brasil de 1996 a 2016: 21 Anos de Contrastes nas Regiões Brasileiras

**DOI:** 10.36660/abc.20190438

**Published:** 2020-11-01

**Authors:** Letícia de Castro Martins Ferreira, Mário Círio Nogueira, Marilia Sá Carvalho, Maria Teresa Bustamante Teixeira

**Affiliations:** 1 Universidade Federal de Juiz de Fora Faculdade de Medicina Juiz de ForaMG Brasil Universidade Federal de Juiz de Fora - Faculdade de Medicina, Juiz de Fora, MG – Brasil; 2 Fundação Oswaldo Cruz Rio de JaneiroRJ Brasil Fundação Oswaldo Cruz Programa de Computação Científica – PROCC, Rio de Janeiro, RJ – Brasil

**Keywords:** Infarto do Miocárdio/mortalidade, Epidemiologia, Mortalidade, Estudos de Séries Temporais, Isquemia Miocárdica

## Abstract

**Fundamento:**

O infarto agudo do miocárdio (IAM), principal causa de morte no Brasil, apresenta disparidades regionais nas tendências temporais das taxas de mortalidade dos últimos anos. Estudos anteriores de tendências temporais não fizeram correção para os códigos-lixo de causas de mortalidade, o que pode ter enviesado as estimativas.

**Objetivo:**

Analisar as desigualdades regionais e por sexo na tendência de mortalidade por IAM no Brasil no período de 1996 a 2016.

**Métodos:**

Estudo de séries temporais de 21 anos (1996 a 2016). Os dados são do Sistema de Informações sobre Mortalidade (SIM) e das estimativas populacionais do Instituto Brasileiro de Geografia e Estatística (IBGE). Foram feitas correções de óbitos por causas mal definidas, códigos-lixo e sub-registro. As séries temporais desagregadas por grandes regiões, sexo, capitais e interior foram analisadas utilizando a técnica de regressão linear segmentada por Jointpoint. O nível de significância estatística adotado foi de 5%.

**Resultados:**

No período, a mortalidade diminuiu mais acentuadamente no sexo feminino (–2,2%; IC 95%: –2,5; –1,9) do que no masculino (–1,7%; IC 95%: –1,9; –1,4), e mais nas capitais (–3,8%; IC 95%: –4,3; –3,3) do que no interior (–1,5%; IC 95%: –1,8; –1,3). Foram verificadas desigualdades regionais com aumento para homens residentes no interior do Norte (3,3; IC 95%: 1,3; 5,4) e Nordeste (1,3%; IC 95%: 1,0; 1,6). O nível de significância estatística adotado foi de 5%. As taxas de mortalidade após correções, principalmente pela redistribuição dos códigos-lixo, apresentaram expressiva diferença em relação às estimativas sem correções.

**Conclusões:**

Embora a mortalidade por IAM apresente redução no Brasil nos últimos anos, essa tendência é desigual segundo região e sexo. Desse modo, as correções dos números de óbitos são essenciais para estimativas mais fidedignas.

## Introdução

Nas últimas décadas, as doenças cardiovasculares (DCV), especificamente a doença isquêmica do coração (DIC), tornaram-se as principais causas de morte no mundo, embora as taxas de mortalidade padronizadas por idade tenham diminuído.^1^

Em estudos sobre mortalidade é importante observar a qualidade dos registros de óbitos, que no Brasil é diferente entre as regiões brasileiras e entre os municípios, sendo melhor nas capitais. Alguns indicadores indiretos da qualidade padrão dos dados são: proporção de óbitos por causas mal definidas, uso de códigos-lixo, sub-registro, idade e sexo ignorados. Eles refletem as dificuldades de diagnóstico das doenças que causaram óbito, de acesso a serviços de saúde, de preenchimento do atestado de óbito e/ou digitação dos dados no sistema.^2^ Um modo de minorar esse problema e estimar adequadamente taxas de mortalidade é efetuar correções para possibilitar maior comparabilidade entre regiões ao longo dos anos.^1^^,^^3^^,^^4^

Este estudo tem por objetivo analisar as desigualdades regionais e por sexo na tendência de mortalidade por IAM no Brasil no período de 1996 a 2016, fazendo a correção dos óbitos por causas mal definidas, códigos-lixo e sub-registro.

## Métodos

Foram analisadas séries temporais (21 anos: 1996 a 2016) de mortalidade por IAM nas capitais e no interior (demais municípios) das grandes regiões brasileiras. Os dados anuais de óbitos por IAM (código I21, CID-10) foram obtidos do SIM e da página eletrônica do Departamento de Informática do Sistema Único de Saúde (Datasus) (http://datasus.saude.gov.br), e as estimativas populacionais, do IBGE. Sendo dados secundários disponíveis publicamente, a pesquisa foi dispensada de aprovação por comitê de ética em pesquisa, conforme resolução do CONEP nº 510, de 07/04/2016. O SIM tem cobertura nacional e, nos últimos anos, melhorou sua qualidade de maneira diferenciada entre regiões e cidades brasileiras. Para possibilitar maior comparabilidade entre regiões e ao longo dos anos, os números obtidos do SIM foram corrigidos quanto a causas mal definidas, uso de códigos-lixo e sub-registro, utilizando procedimentos adotados em outras pesquisas.^1^^,^^3^^,^^5^

**Figura 1:**
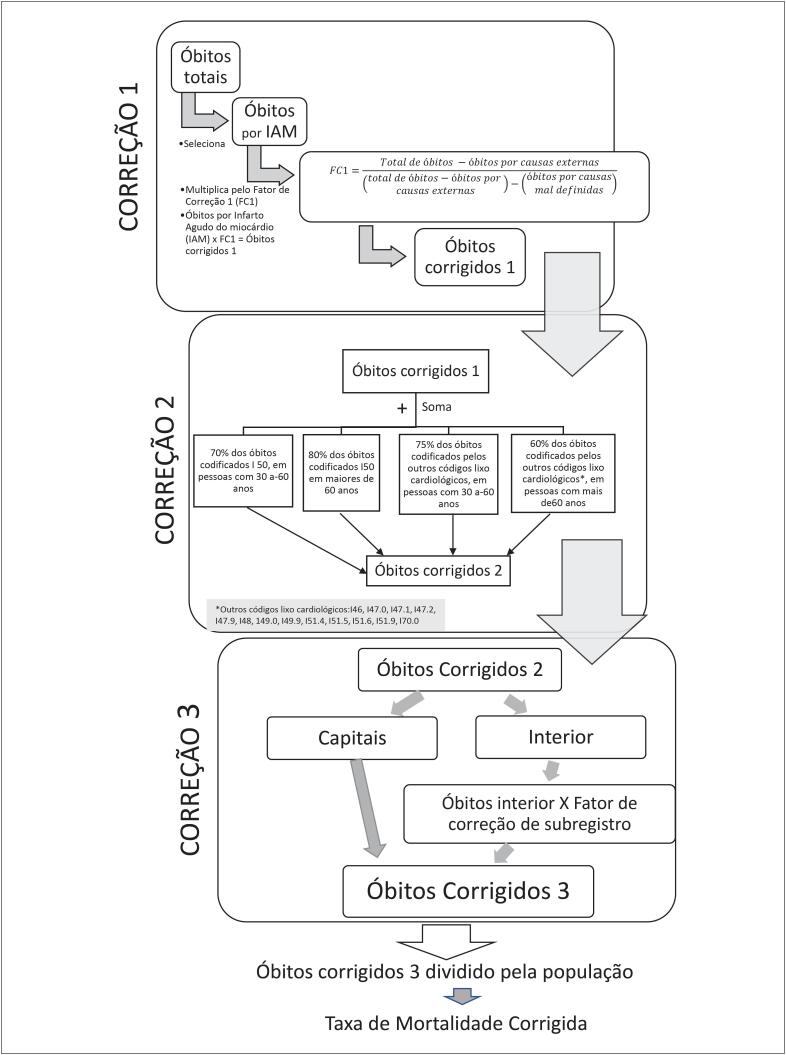
Procedimentos das correções dos números de óbitos quanto a causas mal definidas, uso de códigos-lixo e sub-registro.

Na Figura 1, foram esquematizados os procedimentos de correções dos óbitos por IAM para causas mal definidas, uso de códigos-lixo e sub-registro.

Óbitos por causa mal definida são aqueles em que a causa da morte não foi estabelecida e que são, portanto, classificados nos códigos R00-R99 do Capítulo XVIII da CID-10: “Sintomas, sinais e achados anormais de exames clínicos e de laboratório não classificados em outra parte.”^2^^,^^5^^,^^6^ A redistribuição das causas mal definidas de óbito (correção 1) foi feita do seguinte modo: para cada ano e região, foram calculados fatores de correção (FC1) com a equação (1), para cada sexo e faixa etária de cinco anos. Para a redistribuição dos óbitos, multiplicou-se o seu número pelo FC1.^3^^,^^7^

$$\begin{array}{l} \left(FC1\right)=\frac{\;Total\;de\;óbitos-óbitos\;por\;causas\;externas\;}{(total\;de\;óbitos-óbitos\;por\;causas\;externas)-\;óbitos\;por\;causas\;mal\;definidas} \end{array}$$

Óbitos com códigos-lixo são aqueles em que são usados códigos da CID-10 que são inespecíficos e não caracterizam precisamente a causa básica de óbito.^5^ Em cardiologia, são considerados os seguintes códigos-lixo: I50, I46, I47.0, I47.1, I47.2, I47.9, I48, 149.0, I49.9, I51.4, I51.5, I51.6, I51.9, I70.0. A correção 2 por códigos-lixo foi feita somando os óbitos com códigos-lixo cardiológicos aos óbitos registrados como IAM na seguinte proporção: 70% dos óbitos por I50 em pessoas entre 30 e 60 anos e 80% para pessoas acima de 80 anos, e as demais causas 75% (30 a 60 anos) e 60% (maiores de 60 anos).^5^

Para a correção por sub-registro do óbito (correção 3), ou seja, os óbitos que não foram registrados no Sistema de Registro Civil e no SIM, foram utilizados os fatores de correção estimados para o Brasil, região e estados,^7^ disponíveis no Datasus.^6^ A correção 3 foi feita multiplicando-se o fator de correção de sub-registro pelos óbitos do interior. Para capitais, essa correção não foi realizada, pois estudos mostraram qualidade adequada do registro de óbitos.^2^^,^^3^^,^^5^ Nos anos de 1996 a 1999 e de 2014 a 2016, nos quais não havia disponibilidade dos fatores de correção, foram utilizados valores referentes aos anos mais próximos.

Correções por sexo e idade ignoradas não foram realizadas nesta pesquisa, pois apresentaram bom padrão de preenchimento no período estudado.^4^

As taxas de mortalidade foram calculadas e padronizadas por grupos etários de 5 em 5 anos, para os adultos (20 anos ou mais), usando como referência a nova população-padrão mundial,^8^ que foi proposta pela Organização Mundial da Saúde (OMS) para comparação das taxas de mortalidade entre populações com composições etárias distintas. As estimativas são preparadas para cada ano quinquenal de 1950 a 2025, com base em censos populacionais e outras fontes demográficas, ajustadas para erros de enumeração. A partir dessas estimativas foi construída uma estrutura etária média da população mundial. Nessa pesquisa, as taxas de mortalidade específicas por faixas etárias foram aplicadas sobre os respectivos contingentes populacionais da população-padrão. Consequentemente, obteve-se o número de óbitos esperados para ocorrer em cada faixa etária, caso a população estudada tivesse a mesma composição etária da população-padrão. Dividindo-se o total de óbitos esperados pela população-padrão, obteve-se a taxa de mortalidade padronizada, que pôde ser comparada à de outras populações. As diferenças encontradas não são decorrentes das variações da estrutura etária.^8^^,^^9^

A análise de tendência temporal das taxas corrigidas de mortalidade padronizadas por região, capitais e interior e sexo foi realizada por regressão linear segmentada, utilizando o programa Joinpoint v. 4.6.0.0,^10^^,^^11^ método utilizado em outros estudos de tendência temporal em IAM.^12^^,^^13^ Foram ajustados modelos com pontos de mudança na evolução temporal das taxas (*joint points*), variando de zero (tendência representada por um único segmento de reta) até três. Calcularam-se variações percentuais anuais (APC) para o período estudado. O nível de significância estatística adotado de 5%.

## Resultados

**Figura 2:**
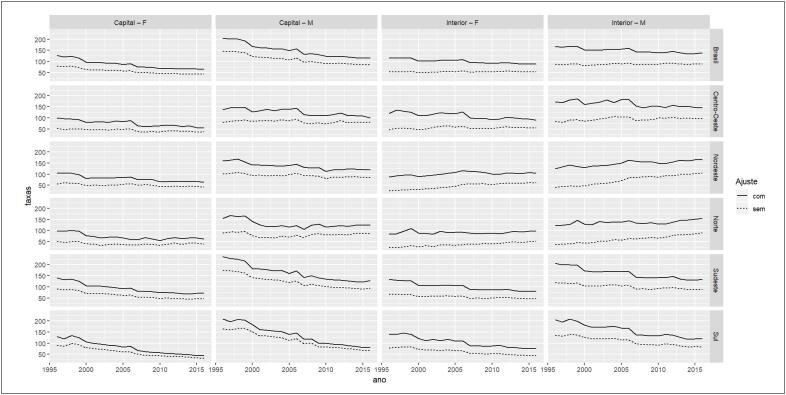
Série temporal após correções de mortalidade por infarto agudo do miocárdio no Brasil, regiões, capitais e interior, por sexo, de 1996 a 2016.

A comparação das taxas de mortalidade por IAM nas regiões do Brasil sem e com correções por causas mal definidas (correção 1), códigos-lixo (correção 2) e sub-registro (correção 3) por sexo feminino e masculino são mostradas nas Tabelas 1 e 2, respectivamente. Maiores aumentos ocorreram após correção pelos códigos-lixo, chegando à elevação de 92% entre mulheres residentes no interior da região Centro-oeste, no ano de 1996. A diferença proporcional das taxas sem e com correções de 1996 a 2016 mostrou pouca discrepância entre estimativas nas capitais. No interior, entretanto, observou-se uma disparidade importante entre elas (Tabela 2). A Figura 2 mostra que não apenas a magnitude das taxas de mortalidade aumenta após correções, mas também a inclinação das tendências temporais se modifica e percentuais de correção são maiores no início do período em relação ao final. A Tabela 3 mostra aumento na frequência dos códigos-lixo nas regiões brasileiras.

**Tabela 1:** Comparação das taxas de mortalidade por infarto agudo do miocárdio (IAM) nas regiões brasileiras com e sem correções por causas mal definidas, códigos-lixo e sub-registro no sexo feminino

Região	Local	Ano	Taxas padronizadas				Mudanças %			
			Não corrigidas	Correção 1	Correção 2	Correção 3	Mudança % 1	Mudança % 2	Mudança % 3	Mudança % total
Brasil	Capital	1996	79,1	82,9	127,1	127,1	5	53	0	61
		2016	42,7	43,7	65,2	65,2	2	49	0	53
		Dif %	–46	–47	–49	–49				
	Interior	1996	52,9	64,3	105,4	116,5	21	64	11	120
		2016	53,6	56,2	85,6	89,4	5	52	4	67
		Dif %	1	–12	–19	–23				
Norte	Capital	1996	50,1	57,6	98,7	98,7	15	71	0	97
		2016	38,1	40,9	62,7	62,7	7	53	0	64
		Dif %	–24	–29	–36	–36				
	Interior	1996	22,5	37,6	57,7	83,4	67	54	45	271
		2016	51,4	55,4	83,1	97,7	8	50	18	90
		Dif %	129	47	44	17				
Nordeste	Capital	1996	56,0	59,1	105,5	105,5	6	79	0	88
		2016	42,5	43,8	63,8	63,8	3	46	0	50
		Dif %	–24	–26	–40	–40				
	Interior	1996	25,4	47,5	68,4	87,9	87	44	28	246
		2016	60,2	64,6	95,7	104,2	7	48	9	73
		Dif %	137	36	40	19				
Centro-oeste	Capital	1996	52,4	55,2	99,5	99,5	5	80	0	90
		2016	38,5	38,8	54,1	54,1	1	39	0	41
		Dif%	−27	−30	−46	−46				
	Interior	1996	46,6	54,8	105,2	120,4	18	92	14	158
		2016	54,4	55,6	83,1	90,0	2	49	8	66
		Dif%	17	2	−21	−25				
Sudeste	Capital	1996	91,7	95,7	140,1	140,1	4	46	0	53
		2016	46,4	47,2	72,5	72,5	2	54	0	56
		Dif %	–49	–51	–48	–48				
	Interior	1996	66,4	74,0	128,0	134,0	11	73	5	102
		2016	47,9	50,4	79,7	81,8	5	58	3	71
		Dif %	–28	–32	–38	–39				
Sul	Capital	1996	91,9	92,2	130,3	130,3	0	41	0	42
		2016	32,3	32,8	44,1	44,1	2	34	0	37
		Dif %	–65	–64	–66	–66				
	Interior	1996	78,4	86,7	136,4	141,9	11	57	4	81
		2016	44,1	45,4	74,2	76,9	3	63	4	74
		Dif %	–44	–48	–46	–46				

Correção 1: taxas de mortalidade por IAM corrigidas por causas mal definidas. Correção 2: taxas de mortalidade por IAM corrigidas por códigos-lixo. Correção 3: taxas de mortalidade por IAM corrigidas por sub-registro.

**Tabela 2:** Comparação das taxas de mortalidade por infarto agudo do miocárdio (IAM) nas regiões brasileiras com e sem correções por causas mal definidas, códigos-lixo e sub-registro no sexo masculino

Região	Local	Ano	Taxas padronizadas				Mudanças %			
			Não corrigidas	Correção 1	Correção 2	Correção 3	Mudança % 1	Mudança % 2	Mudança % 3	Mudança % total
Brasil	Capital	1996	145,4	153,0	205,1	205,1	5	34	0	41
		2016	86,0	88,9	117,5	117,5	3	32	0	37
		Dif %	–41	–42	–43	–43				
	Interior	1996	86,5	105,2	150,2	167,3	22	43	11	93
		2016	89,4	95,8	131,7	138,4	7	37	5	55
		Dif %	3	–9	–12	–17				
Norte	Capital	1996	88,6	105,5	154,5	154,5	19	46	0	74
		2016	85,3	92,7	123,3	123,3	9	33	0	44
		Dif %	–4	–12	–20	–20				
	Interior	1996	37,5	62,8	85,9	122,8	68	37	43	228
		2016	88,3	96,7	131,2	153,6	10	36	17	74
		Dif %	136	54	53	25				
Nordeste	Capital	1996	101,2	107,1	160,6	160,6	6	50	0	59
		2016	84,9	88,0	119,3	119,3	4	36	0	41
		Dif %	–16	–18	–26	–26				
	Interior	1996	39,9	71,8	95,8	123,9	80	33	29	210
		2016	104,2	113,7	152,6	166,4	9	34	9	60
		Dif %	161	58	59	34				
Centro-oeste	Capital	1996	79,0	84,6	136,6	136,6	7	61	0	73
		2016	79,5	80,9	99,9	99,9	2	23	0	26
		Dif %	1	–4	–27	–27				
	Interior	1996	82,9	99,8	154,1	172,8	20	54	12	109
		2016	95,8	99,7	134,5	146,0	4	35	8	52
		Dif %	16	0	–13	–16				
Sudeste	Capital	1996	174,7	182,5	235,9	235,9	4	29	0	35
		2016	92,9	95,8	128,0	128,0	3	34	0	38
		Dif %	–47	–48	–46	–46				
	Interior	1996	118,5	134,1	195,1	206,1	13	46	6	74
		2016	88,5	94,7	129,9	133,2	7	37	3	50
		Dif %	–25	–29	–33	–35				
Sul	Capital	1996	164,6	165,6	208,9	208,9	1	26	0	27
		2016	67,1	68,5	82,3	82,3	2	20	0	23
		Dif %	–59	–59	–61	–61				
	Interior	1996	134,4	149,4	203,7	204,8	11	36	1	52
		2016	83,4	86,7	120,7	121,3	4	39	0	45
		Dif %	–38	–42	–41	–41				

Correção 1: taxas de mortalidade por IAM corrigidas por causas mal definidas. Correção 2: taxas de mortalidade por IAM corrigidas por códigos-lixo. Correção 3: taxas de mortalidade por IAM corrigidas por sub-registro.

**Tabela 3:** Frequência de óbitos classificados com códigos-lixo para infarto agudo do miocárdio (IAM) por ano, sexo e região no Brasil de 1996 a 2016

Ano	Norte		Nordeste		Centro-oeste		Sudeste		Sul		Total		Geral
	M	F	M	F	M	F	M	F	M	F	M	F	
1996	883	813	4.865	5.117	1.670	1.501	14.198	15.996	4.429	5.140	26.045	28.567	54.612
1997	911	797	5.056	5.175	1.816	1.696	13.429	15.240	4.181	4.881	25.393	27.789	53.182
1998	972	898	5.547	5.540	1.758	1.594	13.342	15.350	4.507	5.221	26.126	28.603	54.729
1999	1.043	863	5.379	5.367	1.827	1.492	12.632	14.569	4.098	4.691	24.979	26.982	51.961
2000	1.046	821	5.402	5.431	1.674	1.511	12.138	13.859	4.157	4.837	24.417	26.459	50.876
2001	1.194	919	5.677	5.678	1.770	1.566	11.931	13.603	3.926	4.563	24.498	26.329	50.827
2002	1.103	900	5.783	6.109	1.963	1.668	11.649	13.821	3.915	4.784	24.413	27.282	51.695
2003	1.184	1.002	5.871	6.141	1.974	1.623	12.100	14.002	4.047	4.703	25.176	27.471	52.647
2004	1.183	935	6.484	6.644	1.967	1.700	12.798	14.552	4.196	4.864	26.628	28.695	55.323
2005	1.248	1.051	6.915	7.299	2.092	1.649	12.642	14.131	4.019	4.712	26.916	28.842	55.758
2006	1.329	1.008	8.210	8.463	2.104	1.870	13.354	15.142	4.009	4.939	29.006	31.422	60.428
2007	1.420	1.111	8.469	8.733	2.129	1.830	13.486	15.326	4.316	5.161	29.820	32.161	61.981
2008	1.454	1.205	8.541	8.888	2.125	1.849	13.784	15.670	4.379	5.207	30.283	32.819	63.102
2009	1.464	1.182	8.538	9.017	2.107	1.868	13.588	15.727	4.464	5.223	30.161	33.017	63.178
2010	1.551	1.237	8.300	8.616	2.179	1.965	14.372	16.825	4.464	5.394	30.866	34.037	64.903
2011	1.587	1.405	8.784	9.292	2.128	1.907	14.522	17.280	4.649	5.834	31.670	35.718	67.388
2012	1.611	1.369	8.583	9.038	2.155	2.072	14.340	16.909	4.514	5.302	31.203	34.690	65.893
2013	1.699	1.428	8.923	9.437	2.199	1.994	14.720	16.875	4.873	5.556	32.414	35.290	67.704
2014	1.737	1.458	8.609	9.238	2.197	2.085	14.589	17.028	4.709	5.564	31.841	35.373	67.214
2015	1.843	1.506	9.006	9.925	2.191	2.045	15.143	18.112	4.810	5.615	32.993	37.203	70.196
2016	1.866	1.586	9.200	9.829	2.013	1.770	16.496	18.841	5260	6.063	34.835	38.089	72.924

F: feminino; M: masculino.

**Figura 3:**
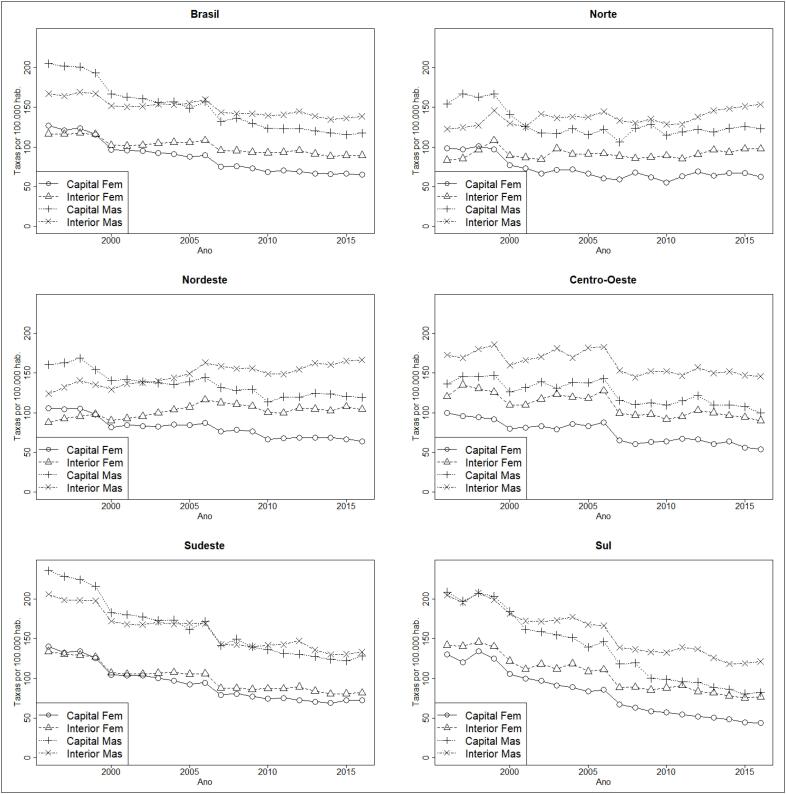
Tendências temporais das taxas de mortalidade por infarto agudo do miocárdio antes (linha tracejada) e após correções por causas mal definidas, sub-registro e códigos-lixo (linha contínua) no Brasil, regiões, capitais e interior, por sexo, de 1996 a 2016.

De maneira geral, as tendências das taxas de mortalidade por IAM corrigidas apresentam declínio. Capitais com maior mortalidade no início do período apresentaram um declínio mais acentuado e, consequentemente, menores taxas nos últimos anos. As taxas de mortalidade do sexo masculino são superiores às do sexo feminino em todo o período analisado, ambas em queda. Somente taxas de mortalidade de homens residentes no interior das regiões Norte e Nordeste apresentaram tendências ao aumento. No início da série, as taxas eram maiores nas regiões Sudeste e Sul e, devido ao declínio mais acentuado nessas regiões, elas passaram a ter valores menores ao final do período do que nas regiões Norte e Nordeste (Figura 3). Houve diferença percentual entre 1996 e 2016 no Brasil, de –43,6%, sendo maior na região Sul (–85,1%). As regiões Nordeste e Norte, que em 1996 apresentavam menores taxas, em 2016 passaram a ter as maiores (Tabela 4).

**Tabela 4:** Taxas de mortalidade* padronizadas pela nova população mundial de infarto agudo do miocárdio (IAM) nas regiões brasileiras nos anos de 1996 e 2016.

Região	1996	2016	Diferença %
Brasil	149,86	104,35	–43,6
Norte	107,05	112,48	4,8
Nordeste	107,42	121,30	11,4
Centro-oeste	135,32	100,47	–34,7
Sudeste	172,93	102,92	–68,0
Sul	168,74	91,14	–85,1

* Por 100.000 habitantes.

A análise de regressão segmentada indica declínio da mortalidade em todas as regiões, com exceção do Nordeste. As regiões Sul (APC = –3,4%; IC 95%: –3,8; –3,0) e Sudeste (APC = –3,3%; IC 95%: –3,9; –2,7) apresentaram maior percentual de decréscimo, e a região Norte, o menor (APC = –0,8%; IC 95%: –1,3; –0,2). Observou-se ainda um padrão diferenciado entre capitais e interior e entre sexos. Houve queda nas taxas de mortalidade por IAM em todas as capitais, bem como no interior das regiões Sudeste, Sul e Centro-oeste. No interior das regiões Norte e Nordeste, as taxas aumentaram, sendo o maior percentual de aumento entre mulheres residentes na região Nordeste entre 2002 e 2006 (APC = 5,2%; IC 95%: 0,2%; 10,5%) (Tabela 5).

**Tabela 5:** Análise da regressão segmentada da tendência da mortalidade por infarto agudo do miocárdio (IAM) por sexo, capitais e interior das regiões brasileiras, de 1996 a 2016

Região	Município	Sexo	Tendência 1			Tendência 2			Tendência 3			Tendência 4		
			Período	APC	IC 95%	Período	APC	IC 95%	Período	APC	IC 95%	Período	APC	IC 95%
Brasil	Capitais	F	1996 a 2010	–4,1	–4,7; –3,6	2010 a 2016	–1,1	–3,2; + 1,0						
		M	1996 a 2010	–3,5	–4,0; –3,1	2010 a 2016	–1,2	–3,0; +0.6						
	Interior	F	1996 a 2016	–1,4	–1,7; –1,1									
		M	1996 a 2016	–1	–1,3; –0,8									
	Todo	Ambos	1996 a 2016	–1,9	–1,7; –2,2									
Norte	Capitais	F	1996 a 2006	–5,1	–6,6; –3,6	2006 a 2016	0,9	–0,7; 2,5						
		M	1996 a 1999	0,6	–5,6; 7,2	1999 a 2002	–10,8	–23,0; 3,4	2002 a 2016	0,5	0,0; 1,0			
	Interior	F	1996 a 2016	0,20	–0,3; 0,7									
		M	1996 a 2005	1,3	0,3; 2,3	2005 a 2010	–1,8	–5,5; 2,0	2010 a 2016	3,3	1,3; 5,4			
	Todo	Ambos	1996 a 2010	–0,8	–1,3; –0,2	2010 a 2016	2,4	0,2; 4,7						
Nordeste	Capitais	F	1996 a 2000	–5,3	–9,1; –1,2	2000 a 2016	–2,0	–2,5; –1,6						
		M	1996 a 2016	–1,6	–2,0; –1,3									
	Interior	F	1996 a 2002	0,60	–	2002 a 2006	5,20	0,2; 10,5	2006 a 2010	–3,0	–7,6; +1,8	2010 a 2016	0,5	–1,1; 2,1
		M	1996 a 2016	1,30	1,0; 1,6									
	Todo	Ambos	1996 a 2003	0,0	–1,2; 1,3	2003 a 2006	5,2	–4,0; 15,3	2006 a 2010	–2,8	–7,2; 1,8	2010 a 2016	1,0	–0,6; 2,6
Centro-oeste	Capitais	F	1996 a 2016	–2,8	–3,3; –2,2									
		M	1996 a 2016	–1,7	–2,1; –1,2									
	Interior	F	1996 a 2016	–1,8	–2,2; –1,3									
		M	1996 a 2016	–1,0	–1,4; –0,6									
	Todo	Ambos	1996 a 2016	–1,7	–2,1; –1,2									
Sudeste	Capitais	F	1996 a 2010	–4	–5,0; –3,8	2010 a 2016	–0,6	–2,8; 1,6						
		M	1996 a 2001	–5,7	–8,1; –3,2	2001 a 2016	–2,9	–2,5; –3,3						
	Interior	F	1996 a 2001	–5,2	–7,5 –2,8	2001 a 2005	0,10	–5,5; 6,2	2005 a 2008	–6,4	–17,5; 6,1	2008 a 2016	–0,9	0,2; –2,1
		M	1996 a 2016	–2,3	–2,6; –1,9									
	Todo	Ambos	1996 a 2009	–3,3	–3,9; –2,7	2009 a 2016	–1,3	–2,9; 0,4						
Sul	Capitais	F	1996 a 2016	–5,8	–6,2; –5,4									
		M	1996 a 2016	–5,2	–5,5; –4,8									
	Interior	F	1996 a 2016	–3,4	–3,8; –3,0									
		M	1996 a 2016	–2,9	–3,3; –2,5									
	Todo	Ambos	1996 a 2016	–3,4	–3,8; –3,0									

F: feminino; M: masculino; ambos: masculino + feminino; todo: região completa. APC: variações percentuais anuais. IC 95%: intervalo de confiança. Nível de significância estatística: 5%.

Maiores quedas nas taxas de mortalidade por IAM ocorreram entre mulheres moradoras nas capitais, com exceção da região Sudeste, onde foi maior entre homens. O maior decréscimo das taxas foi entre mulheres residentes nas capitais da região Sul de 1996 a 2016 (APC = –5,80%; IC 95%: –6,2%; –5,4%), e os menores decréscimos ocorreram entre homens moradores no interior, exceto no Norte (APC = 1,3%; IC 95%; 0,3; 2,3) e no Nordeste (APC = 1,3%; IC 95%; 1,0; 1,6), onde houve aumento (Tabela 5).

## Discussão

Existem poucos estudos de base nacional que, utilizando os dados do SIM, estimaram taxas padronizadas com uso da população mundial padrão e com correções para proporção de óbitos por causas mal definidas, uso de códigos-lixo e sub-registro.^4^ Essas correções são utilizadas por pesquisas de âmbito mundial.^2^^,^^3^^,^^14^ Neste estudo, observa-se que o número de causas mal definidas vem diminuindo ao longo dos anos estudados, assim como o sub-registro mostrando melhorias na qualidade dos dados.^3^ Essa diminuição ocorreu de maneira diferenciada entre as regiões e entre capitais e cidades do interior. Nas regiões Norte e Nordeste e nas cidades do interior, foi mais recente do que nas outras regiões e capitais. Por outro lado, o uso de códigos-lixo não teve uma redução tão significativa quanto as outras, continuando em magnitude muito alta, com ocorrência significativa em todas as regiões.

O declínio nas tendências das taxas de mortalidade por IAM vem sendo observado no mundo e na maioria das regiões brasileiras,^1^^,^^3^^,^^14^^–^^17^ aqui analisadas pela primeira vez separando capitais e interior. Estudos desenvolvidos no Brasil de séries temporais de mortalidade por DCV analisaram grandes regiões brasileiras e evidenciaram diferenças na mortalidade por doenças crônicas não transmissíveis (DCNT) nas regiões.^3^^,^^16^^–^^19^ Essa diferença se justifica em parte pelo aumento das taxas entre homens residentes no interior de tais regiões.

As discrepâncias encontradas na análise por capitais e interior podem ser explicadas pelas transições demográfica e epidemiológica, e pela implantação de políticas públicas de saúde que ocorreram diferentemente em cada região.^16^^,^^20^ Localidades com maior desenvolvimento socioeconômico tiveram transições demográfica e epidemiológica mais precoces através da urbanização, maior acesso à serviços e envelhecimento da população. Isso levou ao aumento das DCNT e das taxas de mortalidade por IAM. Posteriormente, com a implantação de políticas públicas, as taxas começaram a decrescer.^21^ Isso aconteceu em momentos distintos nas diversas cidades e regiões brasileiras. As regiões Sul e Sudeste tiveram essa transição anteriormente às regiões Norte e Nordeste, e as capitais precederam as cidades do interior.^16^^,^^19^ Isso porque, nas capitais, geralmente há mais recursos de saúde, melhores condições socioeconômicas, melhores indicadores de saúde e melhores registros de óbito. O acesso a serviços de média e alta complexidade é também maior. Portanto, taxas de mortalidade por IAM nas capitais foram menores do que no interior, principalmente no período final estudado. Na década de 1990, a série temporal mostra que as taxas no interior eram menores em algumas regiões e passaram por uma inversão no meio do período.^14^^,^^16^^,^^17^ Há de se considerar a questão do sub-registro, além da falta de acesso a serviços de saúde para diagnóstico e preenchimento correto do atestado de óbito no interior, o que pode estar relacionado ao menor número de casos registrados no período,^20^ o que, em parte, justifica a necessidade das correções realizadas.

É interessante ressaltar a inflexão que essas taxas sofrem a partir do ano 2000 em todas as regiões. No Sudeste, Sul e Centro-oeste, há uma queda maior a partir daquele ano, e nas regiões Norte e Nordeste, um aumento das taxas de mortalidade nas cidades do interior. No início deste século, políticas públicas na área da saúde tiveram ampliação e maior aporte de financiamento, como a Política Nacional de Atenção Básica (PNAB) e a Política Nacional de Atenção à Urgência (PNAU). O Serviço de Atenção Móvel de Urgência (SAMU) foi o primeiro componente da PNAU implantado no país no começo dos anos 2000; posteriormente, vieram incentivos para implantação das Unidades de Pronto Atendimento (UPA).^22^ Concomitantemente, os serviços de atenção básica tiveram uma estruturação ampliada com a implantação da Estratégia de Saúde da Família.^21^

Observam-se dois movimentos que propiciaram a diminuição das taxas. O primeiro refere-se a prevenção, controle e tratamento dos fatores de risco da DIC, com atenção básica de maior acesso e qualidade; o outro foi no transporte e diagnóstico precoce e tratamento das DIC com o SAMU e a UPA. Porém, nas regiões Norte e Nordeste, as taxas nas cidades do interior aumentaram, porque essas regiões, historicamente, eram as que tinham maior número de sub-registro e maiores dificuldades de acesso a serviços de saúde, especialmente no interior.^2^^,^^3^ Diante disso, incentivos financeiros federais para organização da atenção básica e dos serviços de urgência e emergência propiciaram a ampliação dos serviços de saúde e, com isso, melhoras nos diagnósticos e registros das causas dos óbitos. Somados às mudanças no perfil de envelhecimento populacional, esses fatores poderiam explicar o aumento dessas taxas.^22^

Em consonância com dados encontrados na literatura, as taxas de mortalidade por DIC nas mulheres foram menores do que nos homens, e a redução de mortes femininas foi maior.^15^^,^^16^ A proteção cardíaca promovida pelos hormônios femininos (estrogênio) pode contribuir para isso. Ações dos estrogênios no endotélio cardiovascular aumentam a liberação de óxido nítrico, que leva à vasodilatação, regula a produção de prostaglandina e inibe a proliferação de músculo liso, fatores que estão relacionados ao IAM.^23^

As limitações deste estudo são inerentes à utilização de dados secundários, embora a qualidade dos registros de óbitos tenha apresentado melhoras ao longo do período analisado. Além disso, foram realizadas correções que contribuíram para aumentar a validade do estudo. Não foram objeto deste estudo os fatores associados a mortalidade por IAM, como obesidade, tabagismo e hipertensão arterial.^24^ Permeando todos esses fatores estão as condições socioeconômicas e culturais com forte influência na mortalidade, identificadas nas diferenças regionais.^15^

## Conclusões

A evolução da mortalidade por IAM no Brasil, no período de 1996 a 2016, apresenta tendência decrescente, caracterizada por desigualdades e contrastes importantes entre sexos, capitais e interior e regiões. Ressalta-se a importância de proceder à correção dos óbitos (por causas mal definidas, códigos-lixo e sub-registros) para possibilitar a construção de indicadores mais confiáveis, que possibilitariam avaliar adequadamente as tendências de mortalidade.
