# Supplementary material for: Mortalidade por Infarto Agudo do Miocárdio no Brasil de 1996 a 2016: 21 Anos de Contrastes nas Regiões Brasileiras
Source: Arq Bras Cardiol. 2020 Nov 1;115(5):849–59. [Article in Portuguese] doi: 10.36660/abc.20190438 (PMC8452190; doi:10.36660/abc.20190438)
Supplement: Translation

# Translation: Mortality Due to Acute Myocardial Infarction in Brazil from 1996 to 2016: 21 Years of Disparities in Brazilian Regions

**Authors:** Letícia de Castro Martins Ferreira, Mário Círio Nogueira, Marilia Sá Carvalho, Maria Teresa Bustamante Teixeira

**Affiliations:** 1 Juiz de ForaMG Brazil Universidade Federal de Juiz de Fora - Faculdade de Medicina, Juiz de Fora, MG – Brazil; 2 Rio de JaneiroRJ Brazil Fundação Oswaldo Cruz Programa de Computação Científica – PROCC, Rio de Janeiro, RJ – Brazil

**Keywords:** Myocardial Infarction, Epidemiology, Mortality, Time Series Studies, Myocardial Ischemia

## Abstract

**Background:**

Acute myocardial infarction (AMI), the leading cause of death in Brazil, has presented regional disparities in mortality rate time trends in recent years. Previous time trend studies did not correct for cause-of-death garbage codes, which may have skewed the estimates.

**Objective:**

To analyze regional and gender-based inequalities in the AMI mortality trend in Brazil from 1996-2016.

**Methods:**

A 21-year time series study (1996-2016). Data are from the Mortality Information System and population estimates from the Brazilian Institute of Geography and Statistics. Corrections of deaths due to ill-defined causes of death, garbage codes, and underreporting were made. The time series broken down by major geographic regions, gender, capital cities, and other municipalities was analyzed using the linear regression technique segmented by Jointpoint. Statistical significance level was set at 5%.

**Results:**

In the period, mortality decreased more sharply in women (−2.2%; 95% CI: −2.5; −1.9) than in men (−1.7%; 95% CI: - 1.9; −1.4) and more in the capital cities (−3.8%; 95% CI: - 4.3; −3.3) than in other municipalities (−1.5%; 95% CI: - 1.8; −1.3). Regional inequalities were observed, with an increase for men living in other municipalities of the North (3.3; 95% CI: 1.3; 5.4) and Northeast (1.3%; 95% CI: 1.0; 1.6). Statistical significance level was set at 5%. Mortality rates after corrections showed a significant difference in relation to the estimates without corrections, mainly due to the redistribution of garbage codes.

**Conclusions:**

Although AMI-related mortality has decreased in Brazil in recent years, this trend is uneven by region and gender. Correcting the numbers of deaths is essential to obtaining more reliable estimates.

## Introduction

In recent decades, cardiovascular diseases (CVD), and specifically ischemic heart disease (IHD) have become the primary causes of death worldwide, although age-standardized mortality rates have dropped.^1^

When conducting mortality studies, one must to pay attention to the quality of death records which, in Brazil, differs between geographic regions and between municipalities, with it being better in the capital cities. Some indirect indicators of standard data quality are the proportion of deaths from ill-defined causes of death, use of garbage codes, underreporting, and ignored age and gender. They reflect difficulties in diagnosing the diseases that caused death, accessing health services, filling out the death certificate, and/or entering data into the system.^2^ One way to solve this problem and properly estimate mortality rates is to make corrections that will allow greater comparability between regions over time.^1^^,^^3^^,^^4^

This study aims to analyze regional and gender inequalities in the AMI-related mortality trend in Brazil from 1996-2016, correcting deaths from ill-defined causes, garbage codes, and underreporting.

## Methods

Time series (21 years: 1996 to 2016) of AMI-related mortality in the capital cities and cities and towns in the countryside (other municipalities) of large Brazilian regions were analyzed. Annual data on AMI-related deaths (code I21. ICD 10) were obtained from the Mortality Information System (SIM in Portuguese) on the DATASUS website - Department of Informatics of the Unified Health System (http://datasus.saude.gov.br) and population estimates from the Brazilian Institute of Geography and Statistics. As publicly available secondary data, the study was exempted from approval by a research ethics committee in accordance with CONEP Resolution 510 of April 7, 2016. The SIM has national coverage and, in recent years, it has greatly improved the quality of its database between Brazilian regions and municipalities. To make a more meaningful comparison between regions and over the time period covered by this study, we corrected the numbers obtained from the SIM regarding ill-defined causes of death, use of garbage codes, and underreporting using procedures adopted in other studies.^1^^,^^3^^,^^5^

Figure 1 outlines the procedures used to correct AMI-related deaths for ill-defined causes, use of garbage codes, and underreporting.

**Figure 1:** Procedures for the correction of the number of deaths regarding ill-defined causes, use of garbage codes, and subregisters.

The ill-defined causes of death are those in which the cause of death has not been established and have therefore been classified in codes R00-R99 from Chapter XVIII of ICD-10: “Unclassified Symptoms, Signs, and Abnormal Clinical and Laboratory Findings Not Elsewhere Classified”.^2^^,^^5^^,^^6^ The redistribution of ill-defined causes of death (Correction 1) was conducted in the following manner: for each year and region, correction factors (CF1) were calculated using equation (1) for each gender and for each five-year age range. To calculate the redistribution of deaths, the number of deaths was multiplied by the CF1.^3^^,^^7^

$$(FC1)=\frac{\;Total\;deaths-deaths\;by\;external\;causes\;}{(total\;deaths-deaths\;by\;external\;causes)-\;deaths\;by\;ill-defined\;causes\;}$$

Deaths with garbage codes are those in which ICD 10 codes are used that are nonspecific and do not precisely identify the underlying cause of death.^5^ The following garbage codes are used in cardiology: I50, I46, I47.0, I47.1, I47.2, I47.9, I48, I49.0, I49.9, I51.4, I51.5, I51.6, I51.9 and I70.0. To proceed with Correction 2 for garbage codes, deaths with cardiologic garbage codes were added to deaths recorded as being caused by AMI in the following proportion: 70% of deaths per 150 in people between 30-60 years of age and 80% for people over 80 years of age, along with other causes 75% (30-60 years of age) and 60% (older than 60 years of age).^5^

To correct for underreported death (Correction 3), meaning deaths that were neither recorded in the Civil Registry System nor in the SIM, the correction factors estimated for Brazil, region, and states,^7^ which are readily available in DATASUS, were used.^6^ Correction 3 was made by multiplying the underreporting correction factor for deaths in other municipalities. This correction was not carried out in the state capitals, as studies have shown that death records in these cities are reliable.^2^^,^^3^^,^^5^ With respect to the periods from 1996 to 1999 and from 2014 to 2016, for which correction factors were not available, values for the next closest years were used.

Corrections due to ignored gender and age were not conducted in this study, since both categories presented reliable numbers during the studied period.^4^

Mortality rates were calculated and standardized by five-year age groups for adults (20 years of age and over) using the new standard world population as a reference.^8^ The standard world population was proposed by the WHO as a way to compare mortality rates between populations with different age groups. Estimates were prepared for each five-year period from 1950 to 2025 based on population censuses and other demographic sources, then adjusted for enumeration errors. From there, an average age structure of the world population was constructed. The specific mortality rates by age groups were applied to the respective population contingents of the standard population. Consequently, the number of deaths expected to occur in each age group was obtained, provided the population studied had the same age composition as the standard population. The standardized mortality rate was obtained by dividing the total number of deaths expected for the standard population. which can then be compared to other populations, and any differences found are not due to variations in the age structure.^8^^,^^9^

The time trend analysis of the corrected mortality rates as standardized by region, capital cities and other municipalities, and gender was performed by segmented linear regression using Joinpoint version 4.6.0.0,^10^^,^^11^ a method used in other AMI time trend studies.^12^^,^^13^ Models were adjusted with change points in the temporal evolution of the death rates (joint points) ranging from zero (trend represented by a single line segment) to three. Annual percentage changes (APC) were calculated for the period under analysis. Statistical significance level was set at 5%.

## Results

A comparison of AMI-related mortality rates in all regions of Brazil, with and without corrections for ill-defined causes of death (Correction 1), garbage codes (Correction 2), and underreporting (Correction 3) by female and male is shown in Tables 1 and 2. respectively. Larger increases were noted after correction for garbage codes, reaching a 92% increase in 1996 among women living in other municipalities of the Central West. The proportional difference in mortality rates with and without corrections, between 1996 and 2016, showed little discrepancy between estimates in the capital cities. However, relative to other municipalities, an important disparity could be seen between them (Table 2). Figure 2 shows that not only does the magnitude of mortality rates increase after corrections, but the time trend slope also changes and percentages of correction are higher at the beginning of the period than at the end. Table 3 shows an increase in the frequency of garbage codes in Brazilian regions.

**Table 1:** Comparison of AMI-related mortality rates in Brazilian regions with and without corrections for ill-defined causes of death, garbage codes, and underreporting in women

Region	Location	Year	Standardized rates				% Changes			
			Not corrected	Correction 1	Correction 2	Correction 3	% Change 1	% Change 2	% Change 3	Total % change
Brazil	Capital cities	1996	79.1	82.9	127.1	127.1	5	53	0	61
		2016	42.7	43.7	65.2	65.2	2	49	0	53
		Dif%	−46	−47	−49	−49				
	Other municipalities	1996	52.9	64.3	105.4	116.5	21	64	11	120
		2016	53.6	56.2	85.6	89.4	5	52	4	67
		Dif%	1	−12	−19	−23				
North	Capital cities	1996	50.1	57.6	98.7	98.7	15	71	0	97
		2016	38.1	40.9	62.7	62.7	7	53	0	64
		Dif%	−24	−29	−36	−36				
	Other municipalities	1996	22.5	37.6	57.7	83.4	67	54	45	271
		2016	51.4	55.4	83.1	97.7	8	50	18	90
		Dif%	129	47	44	17				
Northeast	Capital cities	1996	56.0	59.1	105.5	105.5	6	79	0	88
		2016	42.5	43.8	63.8	63.8	3	46	0	50
		Dif%	−24	−26	−40	−40				
	Other municipalities	1996	25.4	47.5	68.4	87.9	87	44	28	246
		2016	60.2	64.6	95.7	104.2	7	48	9	73
		Dif%	137	36	40	19				
Central West	Capital cities	1996	52.4	55.2	99.5	99.5	5	80	0	90
		2016	38.5	38.8	54.1	54.1	1	39	0	41
		Dif%	−27	−30	−46	−46				
	Other municipalities	1996	46.6	54.8	105.2	120.4	18	92	14	158
		2016	54.4	55.6	83.1	90.0	2	49	8	66
		Dif%	17	2	−21	−25				
Southeast	Capital cities	1996	91.7	95.7	140.1	140.1	4	46	0	53
		2016	46.4	47.2	72.5	72.5	2	54	0	56
		Dif%	−49	−51	−48	−48				
	Other municipalities	1996	66.4	74.0	128.0	134.0	11	73	5	102
		2016	47.9	50.4	79.7	81.8	5	58	3	71
		Dif%	−28	−32	−38	−39				
South	Capital cities	1996	91.9	92.2	130.3	130.3	0	41	0	42
		2016	32.3	32.8	44.1	44.1	2	34	0	37
		Dif%	−65	−64	−66	−66				
	Other municipalities	1996	78.4	86.7	136.4	141.9	11	57	4	81
		2016	44.1	45.4	74.2	76.9	3	63	4	74
		Dif%	−44	−48	−46	−46				

Correction 1: AMI-related mortality rates corrected for ill-defined causes of death. Correction 2: AMI-related mortality rates corrected for garbage codes. Correction 3: AMI-related mortality rates corrected for underreporting.

**Table 2:** Comparison of AMI-related mortality rates in Brazilian regions with and without corrections for ill-defined causes of death, garbage codes, and underreporting in men

Region	Location	Year	Standardized rates				% Changes			
			Not corrected	Correction 1	Correction 2	Correction 3	% Changes 1	% Changes 2	% Changes3	Total % changes
Brazil	Capital cities	1996	145.4	153.0	205.1	205.1	5	34	0	41
		2016	86.0	88.9	117.5	117.5	3	32	0	37
		Dif%	−41	−42	−43	−43				
	Other municipalities	1996	86.5	105.2	150.2	167.3	22	43	11	93
		2016	89.4	95.8	131.7	138.4	7	37	5	55
		Dif%	3	−9	−12	−17				
North	Capital	1996	88.6	105.5	154.5	154.5	19	46	0	74
		2016	85.3	92.7	123.3	123.3	9	33	0	44
		Dif%	−4	−12	−20	−20				
	Other municipalities	1996	37.5	62.8	85.9	122.8	68	37	43	228
		2016	88.3	96.7	131.2	153.6	10	36	17	74
		Dif%	136	54	53	25				
Northeast	Capital	1996	101.2	107.1	160.6	160.6	6	50	0	59
		2016	84.9	88.0	119.3	119.3	4	36	0	41
		Dif%	−16	−18	−26	−26				
	Other municipalities	1996	39.9	71.8	95.8	123.9	80	33	29	210
		2016	104.2	113.7	152.6	166.4	9	34	9	60
		Dif%	161	58	59	34				
Central West	Capital	1996	79.0	84.6	136.6	136.6	7	61	0	73
		2016	79.5	80.9	99.9	99.9	2	23	0	26
		Dif%	1	−4	−27	−27				
	Other municipalities	1996	82.9	99.8	154.1	172.8	20	54	12	109
		2016	95.8	99.7	134.5	146.0	4	35	8	52
		Dif%	16	0	−13	−16				
Southeast	Capital	1996	174.7	182.5	235.9	235.9	4	29	0	35
		2016	92.9	95.8	128.0	128.0	3	34	0	38
		Dif%	−47	−48	−46	−46				
	Other municipalities	1996	118.5	134.1	195.1	206.1	13	46	6	74
		2016	88.5	94.7	129.9	133.2	7	37	3	50
		Dif%	−25	−29	−33	−35				
South	Capital	1996	164.6	165.6	208.9	208.9	1	26	0	27
		2016	67.1	68.5	82.3	82.3	2	20	0	23
		Dif%	−59	−59	−61	−61				
	Other municipalities	1996	134.4	149.4	203.7	204.8	11	36	1	52
		2016	83.4	86.7	120.7	121.3	4	39	0	45
		Dif%	−38	−42	−41	−41				

Correction 1: AMI-related mortality rates corrected for ill-defined causes of death. Correction 2: AMI-related mortality rates corrected for garbage codes. Correction 3: AMI-related mortality rates corrected for underreporting.

**Figure 2:** Time trends in mortality rates from acute myocardial infarction before (dashed line) and after corrections for ill-defined causes of death, underreporting, and garbage codes (continuous line) in Brazil, regions, capital cities, and other municipalities by gender from 1996 to 2016.

**Table 3:** Frequency of deaths classified with garbage codes for AMI by year/gender/region in Brazil from 1996 to 2016

	North		Northeast		Central West		Southeast		South		Total		
Year	M	F	M	F	M	F	M	F	M	F	M	F	General
1996	883	813	4865	5117	1670	1501	14198	15996	4429	5140	26045	28567	54612
1997	911	797	5056	5175	1816	1696	13429	15240	4181	4881	25393	27789	53182
1998	972	898	5547	5540	1758	1594	13342	15350	4507	5221	26126	28603	54729
1999	1043	863	5379	5367	1827	1492	12632	14569	4098	4691	24979	26982	51961
2000	1046	821	5402	5431	1674	1511	12138	13859	4157	4837	24417	26459	50876
2001	1194	919	5677	5678	1770	1566	11931	13603	3926	4563	24498	26329	50827
2002	1103	900	5783	6109	1963	1668	11649	13821	3915	4784	24413	27282	51695
2003	1184	1002	5871	6141	1974	1623	12100	14002	4047	4703	25176	27471	52647
2004	1183	935	6484	6644	1967	1700	12798	14552	4196	4864	26628	28695	55323
2005	1248	1051	6915	7299	2092	1649	12642	14131	4019	4712	26916	28842	55758
2006	1329	1008	8210	8463	2104	1870	13354	15142	4009	4939	29006	31422	60428
2007	1420	1111	8469	8733	2129	1830	13486	15326	4316	5161	29820	32161	61981
2008	1454	1205	8541	8888	2125	1849	13784	15670	4379	5207	30283	32819	63102
2009	1464	1182	8538	9017	2107	1868	13588	15727	4464	5223	30161	33017	63178
2010	1551	1237	8300	8616	2179	1965	14372	16825	4464	5394	30866	34037	64903
2011	1587	1405	8784	9292	2128	1907	14522	17280	4649	5834	31670	35718	67388
2012	1611	1369	8583	9038	2155	2072	14340	16909	4514	5302	31203	34690	65893
2013	1699	1428	8923	9437	2199	1994	14720	16875	4873	5556	32414	35290	67704
2014	1737	1458	8609	9238	2197	2085	14589	17028	4709	5564	31841	35373	67214
2015	1843	1506	9006	9925	2191	2045	15143	18112	4810	5615	32993	37203	70196
2016	1866	1586	9200	9829	2013	1770	16496	18841	5260	6063	34835	38089	72924

F: Female; M: Male.

In general, trends in corrected AMI-related mortality rates are declining. Capital cities, with higher mortality rates at the beginning of the period, showed a more pronounced decline and, consequently, lower rates in recent years. Mortality rates in men were higher than those in women throughout the analyzed period, with both falling. Only mortality rates of men living in other municipalities of the North and Northeast showed an upward trend. At the beginning of the series, mortality rates were higher in the Southeast and South and, due to the more pronounced decline in these regions, their values began to be lower at the end of the period than in the North and Northeast (Figure 3). A percentage difference of −43.6% was observed between 1996 and 2016 in Brazil, with it being higher in the South (−85.1%). The Northeast and North, which in 1996 had the lowest rates, began to reflect the highest rates in 2016 (Table 4).

**Figure 3:** Time series after mortality corrections for acute myocardial infarction in Brazil, regions, capital cities, and other municipalities by gender from 1996 to 2016.

**Table 4:** AMI-related mortality rates * standardized by the new world population by Brazilian region in 1996 and 2016

Region	1996	2016	% Difference
Brazil	149.86	104.35	−43.6
North	107.05	112.48	4.8
Northeast	107.42	121.30	11.4
Central West	135.32	100.47	−34.7
Southeast	172.93	102.92	−68.0
South	168.74	91.14	−85.1

* per 100,000 inhabitants.

The segmented regression analysis indicated a decline in mortality rates in all regions except the Northeast (Table 5). The South (APC = −3.4%; 95% CI: - 3.8; −3.0) and Southeast (APC = −3.3%; 95% CI: - 3.9; −2.7) showed the highest percentage of decrease and the North, the lowest (APC = −0.8%; 95% CI: - 1.3; −0.2).

**Table 5:** Analysis of the segmented regression of the AMI-related mortality trend by gender, capital cities, and other municipalities of the Brazilian regions, 1996-2016

Region	Municipality	Gender	Trend 1			Trend 2			Trend 3			Trend 4		
			Period	APC	IC95%	Period	APC	IC95%	Period	APC	IC 95%	Period	APC	IC95%
Brazil	Capital cities	F	1996 to 2010	−4.1	−4.7; −3.6	2010 to 2016	−1.1	−3.2; + 1.0						
		M	1996 to 2010	−3.5	−4.0; −3.1	2010 to 2016	−1.2	−3.0; +0.6						
	Other municipalities	F	1996 to 2016	−1.4	−1.7; −1.1									
		M	1996 to 2016	−1	−1.3; −0.8									
	All	Both	1996 to 2016	−1.9	−1.7; −2.2									
North	Capital cities	F	1996 to 2006	−5.1	−6.6; −3.6	2006 to 2016	0.9	−0.7; 2.5						
		M	1996 to 1999	0.6	−5.6; 7.2	1999 to 2002	−10.8	−23.0; 3.4	2002 to 2016	0.5	0.0; 1.0			
	Other municipalities	F	1996 to 2016	0.20	−0.3; 0.7									
		M	1996 to 2005	1.3	0.3; 2.3	2005 to 2010	−1.8	−5.5; 2.0	2010 to 2016	3.3	1.3;5.4			
	All	Both	1996 to 2010	−0.8	−1.3; −0.2	2010 to 2016	2.4	0.2; 4.7						
Northeast	Capital cities	F	1996 to 2000	−5.3	−9.1; −1.2	2000 to 2016	−2.0	−2.5; −1.6						
		M	1996 to 2016	−1.6	−2.0; −1.3									
	Other municipalities	F	1996 to 2002	0.60	−1.0; +2.2	2002 to 2006	5.20	0.2;10.5	2006 to 2010	−3.0	−7.6; +1.8	2010 to 2016	0.5	−1.1; 2.1
		M	1996 to 2016	1.30	1.0; 1.6									
	All	Both	1996 to 2003	0.0	−1.2; 1.3	2003 to 2006	5.2	−4.0; 15.3	2006 to 2010	−2.8	−7.2; 1.8	2010 to 2016	1.0	−0.6; 2.6
Central West	Capital cities	F	1996 to 2016	−2.8	−3.3; −2.2									
		M	1996 to 2016	−1.7	−2.1; −1.2									
	Other municipalities	F	1996 to 2016	−1.8	−2.2; −1.3									
		M	1996 a 2016	−1.0	−1.4; −0.6									
	All	Both	1996 to 2016	−1.7	−2.1; −1.2									
Southeast	Capital cities	F	1996 to 2010	−4	−5.0; −3.8	2010 to 2016	−0.6	−2.8;1.6						
		M	1996 to 2001	−5.7	−8.1; −3.2	2001 to 2016	−2.9	−2.5; −3.3						
	Other municipalities	F	1996 to 2001	−5.2	−7.5 - 2.8	2001 to 2005	0.10	−5.5; 6.2	2005 to 2008	−6.4	−17.5 6.1	2008 to 2016	−0.9	0.2; −2.1
		M	1996 to 2016	−2.3	−2.6; −1.9									
	All	Both	1996 to 2009	−3.3	−3.9; −2.7	2009 to 2016	−1.3	−2.9; 0.4						
South	Capital cities	F	1996 to 2016	−5.8	−6.2; −5.4									
		M	1996 to 2016	−5.2	−5.5; −4.8									
	Other municipalities	F	1996 to 2016	−3.4	−3.8; −3.0									
		M	1996 to 2016	−2.9	−3.3; −2.5									
	All	Both	1996 to 2016	−3.4	−3.8; −3.0									

F: female; M: male; Both=Male+Female. All=entire region. APC: annual percentage changes. 95% CI: confidence interval. Statistical significance level: 5%.

A markedly different pattern was observed between the capital cities and other municipalities and between genders. There was a decrease in AMI-related mortality rates in all capitals, as well as in other municipalities of the Southeast, South, and Mid-West (Table 5). Conversely, the same rates increased in other municipalities of the North and Northeast, with the highest percentage of increase among women living in the Northeast between 2002 to 2006 (APC = 5.2%; 95% CI: 0.2%; 10.5%) (Table 5).

More significant declines in AMI-related mortality rates were observed among women living in the capital cities, except for the Southeast, where it was higher among men. The greatest decrease was observed among women living in the capital cities of the South from 1996 to 2016 (APC = −5.80%; 95% CI: - 6.2%; −5.4%). Smaller decreases were found among men living in other municipalities, except in the North (APC = 1.3%; 95% CI: 0.3; 2.3) and Northeast (APC = 1.3%; 95% CI: 1.0; 1.6), where an increase was observed (Table 5).

## Discussion

Few nationally-based studies using data from the SIM system have attempted to estimate standardized mortality rates by means of the standard world population and with corrections for the proportion of deaths from ill-defined causes of death, the use of garbage codes, and underreporting.^4^ These corrections are used by studies worldwide.^2^^,^^3^^,^^14^ It was observed in this study that the number of ill-defined and underreported causes declined over the years studied, thus indicating improvements in data quality.^3^ This decrease occurred differently between regions and between capital cities and other municipalities. It was more recent in the North and Northeast and in other municipalities than in other regions and capital cities. In contrast, the use of garbage codes showed no signs of significant reduction, remaining very high in number in all regions.

The decline in trends in AMI-related mortality rates has been observed worldwide and in most Brazilian regions,^1^^,^^3^^,^^14^^–^^17^ which is being studied for the first time in this study by capital cities and other municipalities. Time series studies of mortality from cardiovascular diseases developed in Brazil analyzed large regions and found differences in mortality from chronic non-communicable diseases (NCDs) in those regions.^3^^,^^16^^–^^19^ This difference is partly justified by the increase in mortality rates among men living there.

Discrepancies found in the analysis by capital cities and other municipalities can be explained by demographic and epidemiological transitions, as well as the implementation of public health policies that occurred differently in these regions.^16^^,^^20^ Areas with greater socioeconomic development had earlier demographic and epidemiological transitions through urbanization, greater access to services, and the presence of an aging population. This led to a rise in chronic noncommunicable diseases and AMI-related mortality rates. Subsequently, they began to drop as new public policies were implemented.^21^ This transition occurred at different times in different cities and regions. The South and Southeast experienced it before the North and Northeast, even as capital cities preceded other municipalities.^16^^,^^19^ Capital cities generally offer more healthcare resources, better socioeconomic conditions, better health indicators, and better death records. Access to medium- and high-complexity services is also greater. Therefore, AMI-related mortality rates in the capital cities were lower than those in other municipalities, primarily in the final studied period. The time series in the 1990s revealed that mortality rates in other municipalities were lower in some regions and underwent an inversion in the middle of the period.^14^^,^^16^^,^^17^ The issue of underreporting also needs to be taken into account, along with the lack of access to health services for diagnosis and the proper completion of death certificates in other municipalities, which may explain the smaller number of cases recorded in this period^20^ and justifies, in part, the need for the corrections made.

It is interesting to highlight the turning point that these rates have undergone in all regions since 2000. A greater drop was observed in the Southeast, South, and Central West starting that year, whereas an increase in mortality rates was noted in other municipalities in the North and Northeast. This was a time when public policies in the healthcare area began to expand with increased funding, such as the National Primary Care Policy (PNAB in Portuguese) and the National Emergency Care Policy (PNAU in Portuguese). The Mobile Emergency Care Service (SAMU in Portuguese) was the first component of the PNAU to be implemented in the country in the early 2000s.^22^ Later came incentives for the implementation of Emergency Care Units (UPA).^22^ Concomitantly, primary care services wound up with an expanded structure through the implementation of the Family Health Strategy.^21^

Two movements led to a decline in mortality rates: one in relation to the prevention, control, and treatment of risk factors for IHD, with greater access to quality primary care, and another in the transportation, early diagnosis, and treatment of IHD through SAMU and emergency care units (UPA in Portuguese). However, in the North and Northeast, mortality rates rose in other municipalities. Historically, these have been the regions with the highest numbers of underreporting and the most difficulties in accessing healthcare services, especially in other municipalities.^2^^,^^3^ Federal financial incentives aimed at organizing primary care and urgent and emergency services provided them with much-needed expansion in healthcare services and, with that, improvements in diagnoses and records of the causes of deaths, which, when added to changes arising from an aging population, could explain why mortality rates have risen.^22^

IHD mortality rates in women were lower than in men and the reduction in female deaths was also greater, which is in line with data found in the literature.^15^^,^^16^ The cardiac protection promoted by female hormones (estrogen) may contribute to it. The presence of estrogen in the cardiovascular endothelium triggers the release of nitric oxide, leading to vasodilation; regulates prostaglandin production; and inhibits smooth muscle proliferation, factors related to AMI.^23^

The limitations of this study are inherent to the use of secondary data, although the quality of death records did improve during the analyzed period. Moreover, corrections were made that enhanced its validity. The factors associated with AMI-related mortality, such as obesity, smoking, and arterial hypertension, were not the object of this study.^24^ Permeating all of these factors are socioeconomic and cultural conditions that strongly influence the mortality rates identified in regional differences.^15^

## Conclusions

The evolution of AMI-related mortality in Brazil from 1996 to 2016 showed a downward trend, characterized by important inequalities and disparities between genders, capital cities and other municipalities and regions. The importance of correcting causes of death (due to ill-defined causes, garbage codes, and underreporting) was emphasized to encourage the construction of more reliable indicators that would allow a proper assessment of mortality trends to be made.
